# Green Preparation of Antimicrobial 1D-Coordination Polymers: *[Zn(4,4′-bipy)Cl_2_]_∞_* and *[Zn(4,4′-bipy)_2_(OAc)_2_]_∞_* by Ultrasonication of Zn(II) Salts and 4,4′-Bipyridine

**DOI:** 10.3390/molecules27196677

**Published:** 2022-10-07

**Authors:** Alessandra Scano, Elisabetta Mereu, Valentina Cabras, Giada Mannias, Alessandra Garau, Martina Pilloni, Germano Orrù, Alessandra Scano, Guido Ennas

**Affiliations:** 1 Department of Chemical and Geological Sciences, University of Cagliari and INSTM Unit, SS 554 Bivio per Sestu, 09042 Monserrato, CA, Italy; 2 Department of Surgical Sciences, Molecular Biology Service, University of Cagliari, 09124 Cagliari, Italy

**Keywords:** green chemistry, sonochemistry, 4,4′–bipyridine, zinc (II) coordination polymers, X-ray powder diffraction, thermal studies, antimicrobial activity, *Candida* spp., *Klebsiella pneumoniae*, *Staphylococcus aureus*

## Abstract

We report on the green preparation of one-dimensional metal coordination polymers by sonochemical approach. The spacer ligand 4,4′-bipyridine was ultrasonicated with chloride or acetate zinc salts to obtain *[Zn(4,4′-bipy)Cl_2_]_∞_* and *[Zn(4,4′-bipy)_2_(OAc)_2_]_∞_*, respectively. Benign solvents such as ethanol and water were selected as reaction media, and the synthesis took place in a few minutes—a very short time compared to conventional methods where some days’ synthesis is required. X-ray powder diffraction, Fourier transform infrared spectroscopy, thermal analysis (thermogravimetric and differential scanning calorimetry), and CHN techniques investigated the influence of using different reaction solvents on the chemical, structural, and thermal properties of the final products. The 1D *[Zn(4,4′-bipy)Cl_2_]_∞_* and *[Zn(4,4′-bipy)_2_(OAc)_2_]_∞_* polymers, in agreement with the structures reported in the literature, were obtained in the form of nanocrystals with an average crystal size around 100 nm. As a proof of concept, a set of Gram-positive (*Staphylococcus aureus*) and Gram-negative bacteria (*Klebsiella pneumoniae)*, and three yeast strains (*Candida albicans*, *Candida krusei*, *Candida glabrata*) were tested to evaluate the antimicrobial activity of the coordination polymers, following the Kirby–Bauer procedure and microplate dilution method. Thus, minimum inhibitory concentration (MIC), minimum bactericidal concentration (MBC), and minimal biofilm inhibitory concentration (MBIC) were evaluated. Except for *Candida krusei*, the compounds showed an appreciable antimicrobial and antibiofilm activity against these strains grown in the liquid medium.

## 1. Introduction

Coordination polymers (CPs) have received a great research interest in recent years [1] because of their potential applications in catalysis [2,3], magnetism [4,5], optics [6,7], sensing [8,9,10], medical diagnostics [11], energy and data storage [12], host-guest chemistry [13,14], gas capture [15,16], molecular separation [17,18,19,20], and so on [21,22]. However, although these materials offer very interesting properties and could be used in several potential applications, to move from laboratory studies to real technologies, important drawbacks still need to be overcome. In particular, an important point is the scaling-up of the material production towards commercial applications, which should avoid health and environmental hazards, high-cost production, and low sustainability [23,24]. Unfortunately, the use of green chemistry to prepare CPs is still at an early stage [23]. The current, mostly used synthetic approaches include diffusion-based method [25], layering technique, evaporation routes [26], hydrothermal/solvothermal synthesis, [27] and crystallization techniques not suitable for large-scale production. In fact, most of them involve the use of harsh reaction conditions and hazardous organic solvents or, at best, require very long reaction times of several days. Thus, to jump from academic studies to commercialization [11,12], safe production routes with low environmental impact and high-energy efficiency are mandatory.

A green alternative is offered by the ultrasonic irradiation method also known as sonochemistry [28,29,30]. Irradiation of a liquid or liquid–solid slurries with high intensity ultrasonic energy induces acoustic cavitation processes that include formation, growth, and collapse of gas bubbles. The rapid collapse of these gas bubbles creates extreme local conditions of high temperatures (ca. 5000 K) and pressure (ca. 1000 atm) as well as cooling rates above 10^10^ K·s^−1^ [31]. This favors the synthesis of materials often unavailable by conventional methods [32,33]. Sonochemistry allows shorter reaction times of minutes and higher reaction yields in comparison with conventional synthesis such as hydrothermal/solvothermal methods. [34,35] Moreover, sonochemistry does not require pressure control or high temperature, and therefore, it is considered a simple, cost effective, and environmentally friendly approach to synthesize the coordination of supramolecular compounds [36,37,38]. However, despite the abovementioned advantages, the literature reports on several sonochemical processes to obtain CPs that still use unfavourable solvents that have health and environmental risks [23,39].

In this scenario, we propose the preparation of *[Zn(4,4′-bipy)Cl_2_]_∞_* and *[Zn(4,4′-bipy)_2_(OAc)_2_]_∞_* 1 dimensional (1D) CPs by sonochemical process, starting from chloride or acetate zinc (II) salts and 4,4′-bipyridine (4,4′ bipy). Zinc is an important trace element in human organisms, and Zn^2+^ is not hazardous to the environment and to human health metal ions. Moreover, this ion is one of the most suitable metal centres for the construction of CPs because of its spherical d^10^ electronic configuration, which could give rise to flexible and reversible coordination environments [40]. Therefore, the geometries of Zn complexes can vary from 4 to 6 by tetrahedral through trigonal bipyramidal and square pyramidal to octahedral [39]. Moreover, some distortion of the ideal polyhedron can easily occur and, in some dimeric units, Zn^2+^ ions exhibit different coordination [40,41]; 4,4′ bipy is a common ligand used in the preparation of CPs. Due to its rigidity, it allows for some degree of control upon the steric constraints of the assembly process. It can be isolated in the leaves of the common weed *Cosmos caudatus*, and it seems to have a slight antifungal activity against the fungus *Candida albicans* and against *Saccharomyces cerevisiae* [42].

The literature already reports on these zinc (II) CPs containing 4,4′ bipy spacer ligands prepared with conventional chemical techniques [43]. In our study, we have demonstrated the synthesis of two model 1D CPs—*[Zn(4,4′-bipy)Cl_2_]_∞_* and *[Zn(4,4′-bipy)_2_(OAc)_2_]_∞_*—by using a health and eco-friendly process. To introduce sustainability in the design and manufacturing of the CPs, we run the interaction of 4-4′-bipy with the zinc-nodes in ideal solvents such as water, or recyclable ones such as ethanol and methanol and by the application of ultrasound. Our intention was to explore whether the novel synthesis process could lead to different coordination arrays with respect to the conventional ones. Moreover, considering the antimicrobial capability of Zn^2+^ ions and 4,4′ bipy singularly [44], we were also interested in evaluating the antimicrobial activity of the two CPs derived from their combinations. Recently, Colinas and co-workers reported on the antibacterial activity of *[Zn(bipy)(OH_2_)_4_^2+^]_1.5_[ClO_4_)^−^]_3_·(bipy)_3_(H_2_O)* and *[Zn_1.5_(CH_3_CO_2_)_2_(bipy)_2_^+^][ClO_4_)^−^]·(H_2_O)* CPs [45], but to our knowledge, this is the first study that investigates antimicrobial properties of *[Zn(4,4′-bipy)Cl_2_]_∞_* and *[Zn(4,4′-bipy)_2_(OAc)_2_]_∞_* [46].

Thus, the obtained samples were physico-chemically characterized by elemental analysis (CHN), X-ray powder diffraction (XRPD), Fourier transform infrared spectroscopy techniques (FTIR), thermal analysis (thermogravimetric analysis, TG, and differential scanning calorimetry, DSC). Moreover, the antimicrobial activity of the samples was tested against a set of bacteria (*Klebsiella pneumoniae* (DSM 681) and *Staphylococcus aureus* (DSM1104)) and yeast (*Candida albicans* (DSM 1386), *Candida krusei* (DSM 70075) e *Candida glabrata* (DSM 6425)).

## 2. Results and Discussion

### 2.1. [Zn(4,4′-bipy)Cl_2_]_∞_

**Figure 1:**
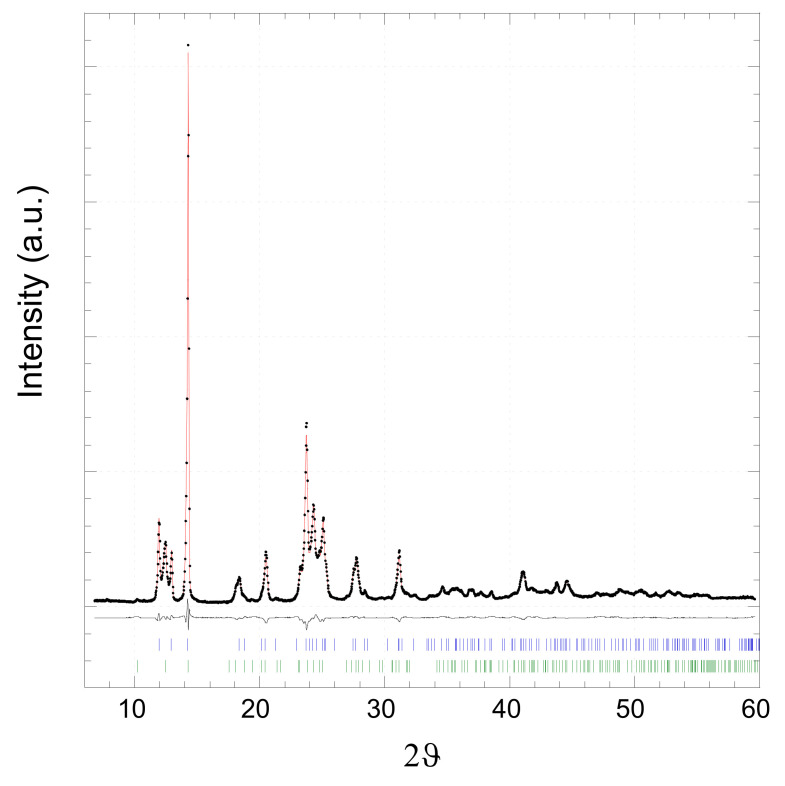
Rietveld refinement of XRPD pattern for the *[Zn(4,4′-bipy)Cl_2_]_∞_* sample: experimental data (black dots), simulated curve (red line), and difference curve (black line). Lower vertical marks represent reflection positions of UBOCIX02 (blue) and UBOCIX03 (green) phase obtained by single-crystal data [47,48].

Figure 1 shows the XRPD pattern of [*Zn(4,4′-bipy)Cl_2_*]*_∞_* sample prepared by the sonochemical process in methanol. Rietveld refinement of the pattern has been carried out with two different polymorphs of [*Zn(4,4′-bipy)Cl_2_*]*_∞_* catena-((μ_2_-4,4′-Bipyridine-N,N′)-dichloro-zinc(II)) [47,48]:

- Polymorph I—UBOCIX02 (a = 15.821(3) Å, b = 5.109(1) Å, c = 14.612(3) Å, b = 110.33(3)°, monoclinic, C2/c);
- Polymorph II—UBOCIX03 (a = 17.349(3) Å, b = 12.407(2) Å, c = 5.114(1) Å, orthorhombic, Pnma.

Both phases reported in the literature have been obtained by hydrothermal/solvothermal methods [47,48]. The fit results are summarized in Table 1. Rietveld refinement indicates that the almost complete conversion of reactants into the coordination polymer [*Zn(4,4′-bipy)Cl_2_*]_∞_ was obtained. Lattice parameters are in agreement with the current literature. The width of the peaks indicates the presence of nanocrystals with an average crystalline size around 100 nm without any strain or stress effects. In the molecular structures of both polymorphic phases [*Zn(4,4′-bipy)Cl_2_*]_∞_ UBOCIX03 and UBOCIX02 (reported in Supplementary Figures S1 and S2), each Zn^2+^ ion is coordinated by two nitrogen atoms from two 4,4-bipy ligands and two chlorine atoms to form a distorted tetrahedral geometry. The dihedral angle of two pyridine rings in the 4,4′-bipy is close to 3.5° indicating that they are not strictly coplanar. The overall coordination type is a one-dimensional zigzag infinite chain. π–π interaction involving two adjacent parallel 4,4′ bipy ligands presents with an average distance of 3.85 Å.

**Figure 2:**
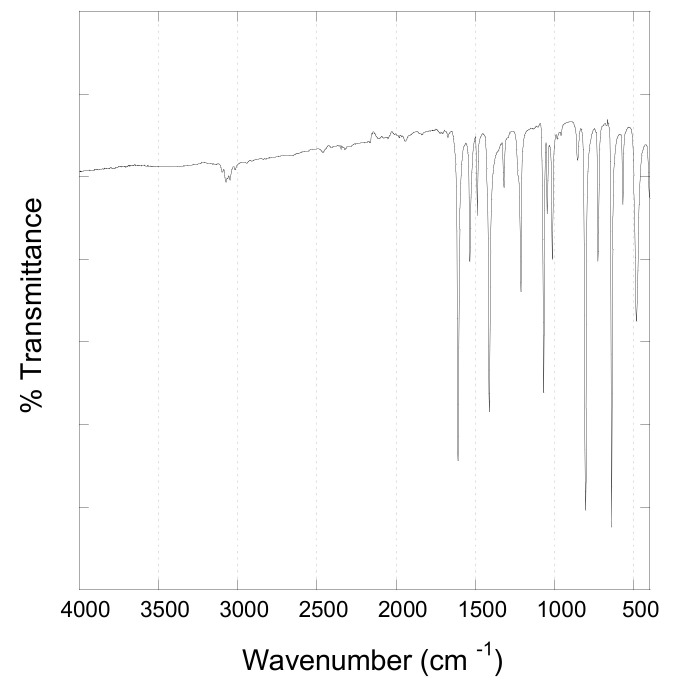
FTIR spectra of [*Zn(4,4′*−*bipy)Cl_2_*]_∞_ sample.

The infrared spectra of the sample *[Zn(4,4′-bipy)Cl_2_]_∞_* (Figure 2) shows peaks at 1613 and 1484 cm^−1^, which are attributed to the ν(C-C) stretching vibration of the aromatic pyridine ring. The peaks at 3052 and 807 cm^−1^ are due to the ν(C-H) vibration of the pyridine ring, and peaks at 1210 and 1226 cm^−1^ are the stretching vibration of the ν(C-N) bond. The most characteristic ring vibration ν(C-N), ν(C-C) and 4,4′bipy breathing modes can be observed in the range 1600.0–1608.9, 1533.4–1537.5 and 1004–1018.3 cm^−1^, respectively. These frequencies are shifted to higher values when compared to the free 4,4′-bipy, indicating Zn coordination which is confirmed by the ν (Zn-N) stretching vibration at 405 cm^−1^ [49].

**Figure 3:**
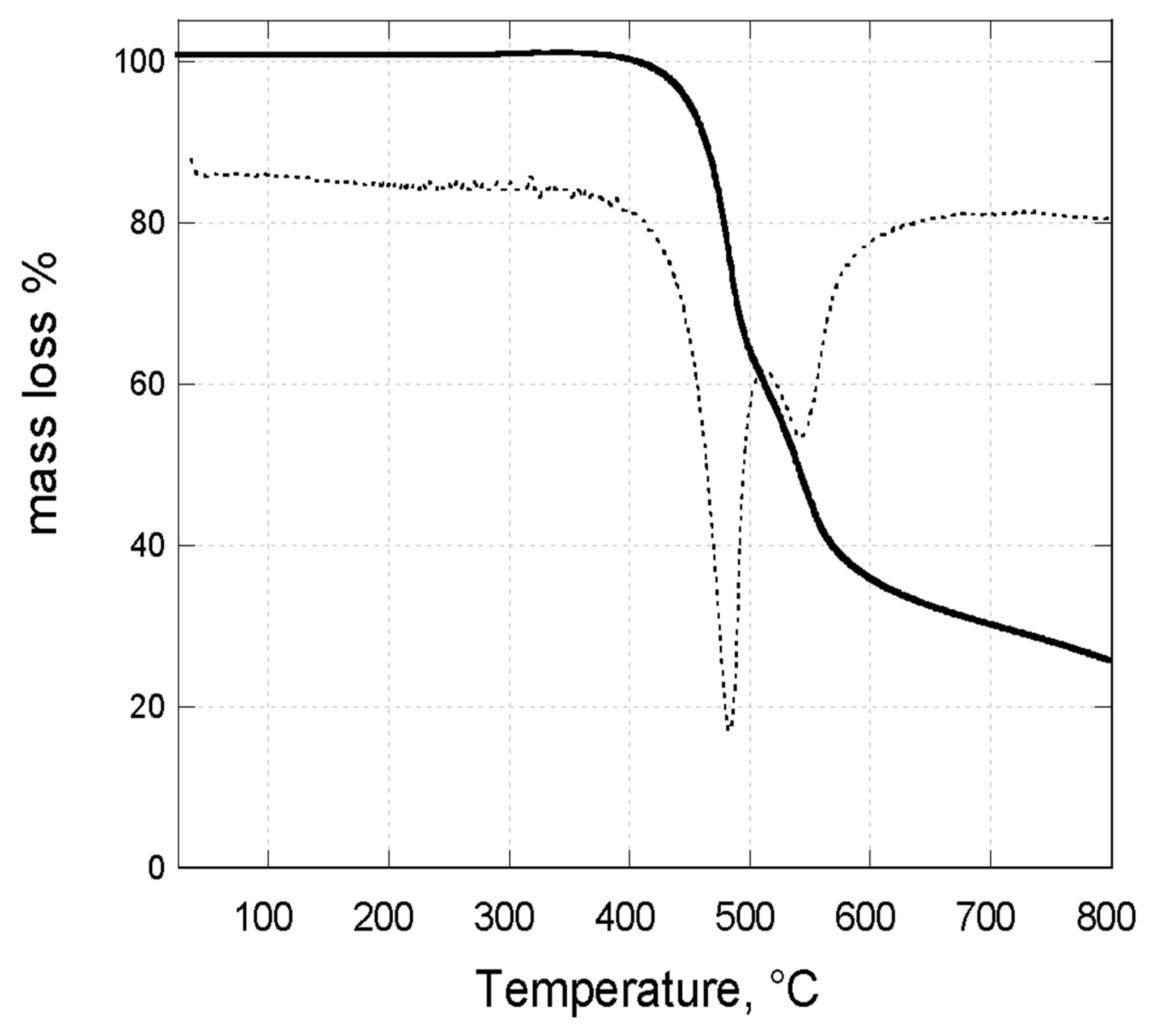
TG (line) and dTG (dashed line) curves of the *[Zn(4,4′-bipy)Cl_2_]_∞_* sample.

**Figure 4:**
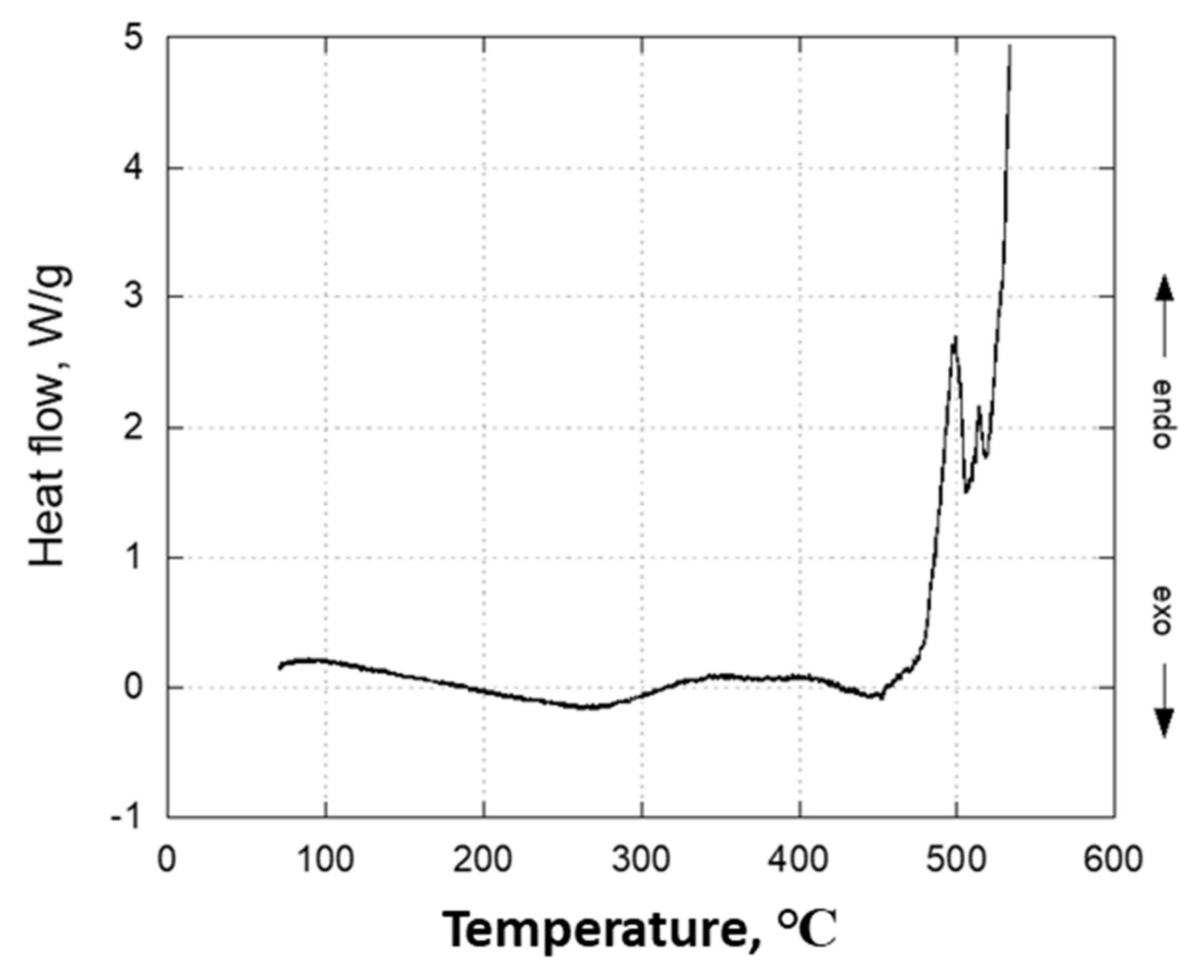
DSC curve of the *[Zn(4,4′*−*bipy)Cl_2_]_∞_* sample.

The thermal behaviour of the [*Zn(4,4′-bipy)Cl_2_*]_∞_ sample prepared by sonochemical method, was observed using thermogravimetric (TG) analysis and differential scanning calorimetry (DSC) measurements (Figure 3 and Figure 4). In the TG and the corresponding derivative (dTG) curve, a main mass loss (and dTG peak) in the temperature range 380–600 °C is observed. A weak shoulder in the range 500–660 °C and a second dTG peak are observed. The steps of the TG curve reflect the almost complete thermal decomposition of the ligands in *[Zn(4,4′-bipy)Cl_2_]_∞_*, with a mass remaining around 25 wt% (theoretical complete decomposition to metallic Zn is 22.4 wt%). XRPD analysis of the remaining sample (not reported) indicates the presence of amorphous Zn-containing carbonaceous residuals confirming the TGA results. In agreement with the TGA, the DSC curve shows a first endothermic peak centred at 500 °C with a second weaker peak at 515 °C, followed by a huge endothermic peak. This behaviour is in agreement with TGA results and indicates the endothermic sample decomposition. In the DSC thermogram no evident signal due to the (I)–(II) polymorphic transition is detected before the decomposition endothermic signal [50].

### 2.2. [Zn(4,4′-bipy)_2_(OAc)_2_]_∞_

**Figure 5:**
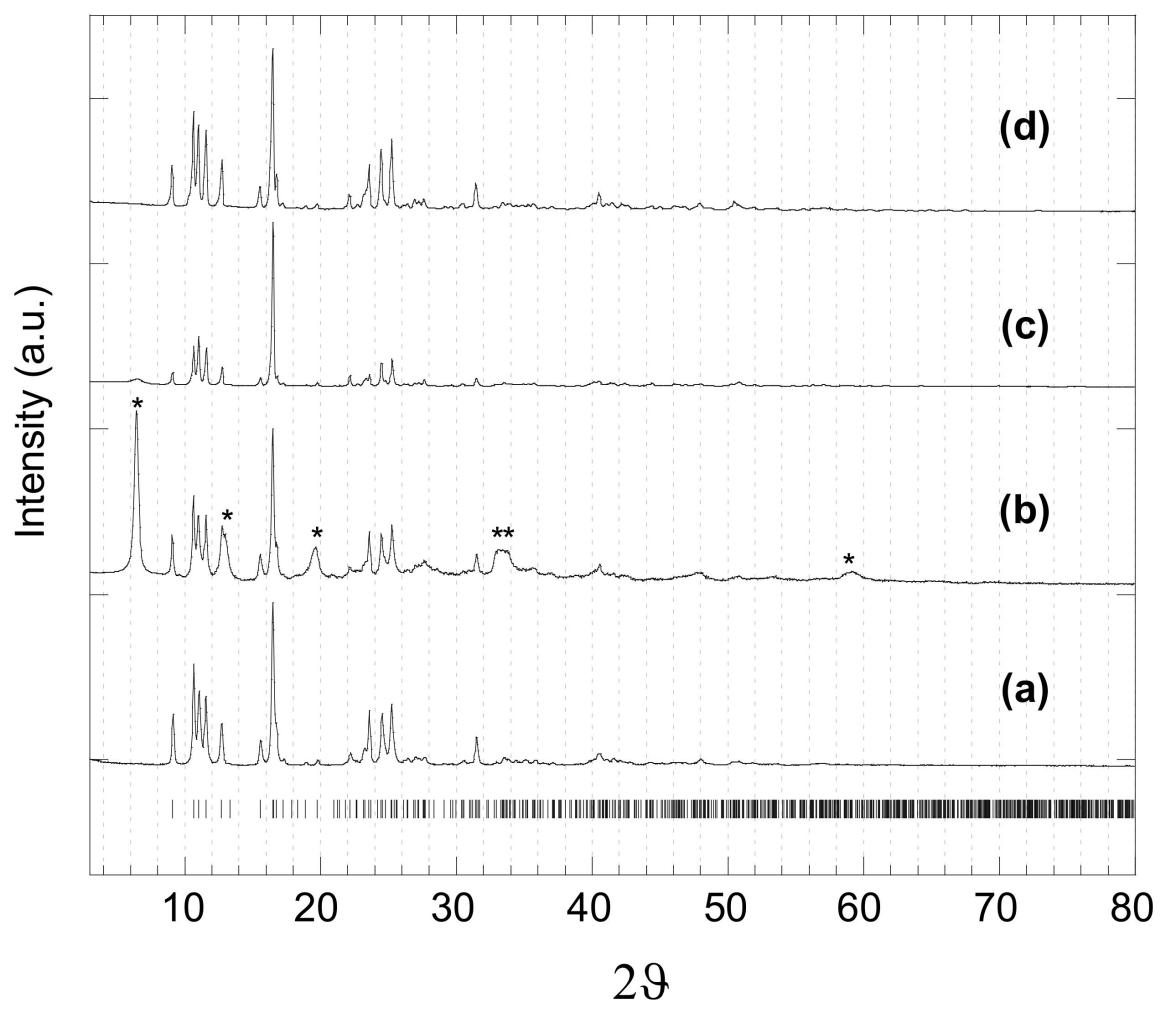
XRPD pattern of *[Zn(4,4′-bipy)_2_(OAc)_2_]_∞_* samples prepared by the sonochemical process: (**a**) sample A (with EtOH-100); (**b**) sample B (with EtOH-96); (**c**) sample C (like sample B but methanol (MeOH) washed; (**d**) sample D (prepared in aqueous solution and washed in methanol). The lower vertical bars represent the reflection position of the *[Zn(4,4′-bipy)_2_(OAc)_2_]_∞_* solvothermal sample reported in the literature [51].

Figure 5 shows the XRPD pattern of *[Zn(4,4′-bipy)_2_(OAc)_2_]_∞_* samples prepared by four different protocols. When the coordination polymer was prepared using absolute ethanol (EtOH-100)/CH_2_Cl_2_ mixture (sample A), the XRPD pattern (Figure 5a) corresponds with the patterns of *[Zn(4,4′-bipy)_2_(OAc)_2_]_∞_* sample reported in the literature and obtained by hydrothermal/solvothermal methods (Table 2). It indicates that the almost complete conversion of reactants into coordination polymer *[Zn(4,4′-bipy)_2_(OAc)_2_]_∞_* was obtained [51]. No peaks due to other Zn-containing phases are detectable [40].

In this structure each Zn^2+^ ion exhibits a quite irregular octahedral geometry (Figure S4). It consists of [Zn(OAc)_2_]_2_ dimeric core, where the acetate ions act as both chelating and bridging ligands. In fact, two acetate ions form a four membered chelate ring (Zn(II)-O-C-O), while other two acetate ions form a bridge connecting two Zn^2+^ ions. Moreover, each Zn^2+^ ion completes the pseudo-octahedral geometry with two nitrogen atoms of two 4,4′-bipy ligands, above and below the dimeric core. The dihedral angle of two pyridine rings in the 4,4′- bipy is 1.7°, indicating that they are almost coplanar. The distance of Zn^2+^ ions in the dimeric unit is 3.95 Å. The overall coordination type is a distinctive one-dimensional double chain structure [41,51,52]. Face-to face π–π interaction involving two adjacent parallel 4,4′ bipy ligands is present with an average distance of 3.60 Å.

The XRPD pattern of sample B prepared using EtOH-96/CH_2_Cl_2_ mixture shows two different phases (Figure 5b). The first phase corresponds to the *[Zn(4,4′-bipy)_2_(OAc)_2_]_∞_* polymeric structure while the second one is an unknown phase (related peaks are indicated by * in Figure 5). This second phase is characterized by a very intense peak at 6.4 ° 2ϑ and other isolated peaks at 13.05, 19.56, 33.15, 33.60, 59.04° 2ϑ. This result agrees with the obtained carbon, hydrogen and nitrogen content for this sample. In fact, the CHN analysis shows a lower carbon, hydrogen and nitrogen content with respect to the pure *[Zn(4,4′-bipy)_2_(OAc)_2_]_∞_* polymer, indicating the presence of a secondary phase with low carbon, hydrogen and nitrogen content. With the aim to purify the desired polymer, sample B was washed with methanol. The XRPD pattern of the obtained sample C (Figure 5d) is very similar to those obtained working with absolute EtOH and corresponds to the pure *[Zn(4,4′-bipy)_2_(OAc)_2_]_∞_* polymer.

**Figure 6:**
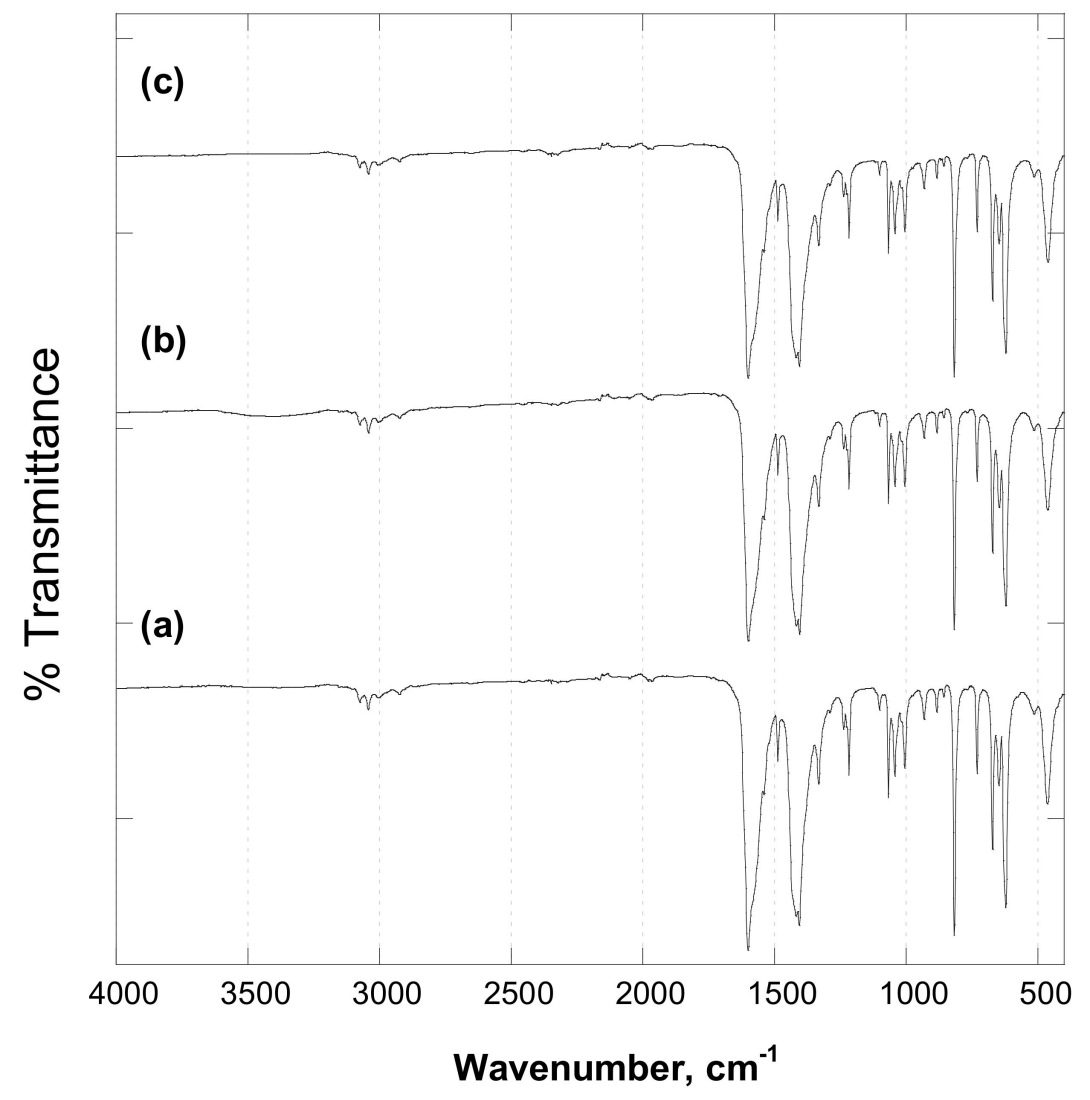
Infrared spectra of *[Zn(4,4′-bipy)_2_(OAc)_2_]_∞_* samples prepared by the sonochemical process: (**a**) sample A; (**b**) sample B; (**c**) sample C.

Figure 6 shows the results of FTIR analysis. The sample A infrared spectra is in agreement with *[Zn(4,4′-bipy)_2_(OAc)_2_]_∞_* molecular structure (Figure 6a). Peaks around 3078 and 2925 cm^−1^ are attributed to the ν(C-H) vibration of the pyridine ring, while peaks at 2854 cm^−1^ are due to the ν(C-H) in the methyl group. Furthermore, the sample shows a peak at 1540 cm^−1^, which is attributed to the ν(C-C) stretching vibration of the aromatic pyridine ring, and a peak at 813 cm^−1^ due to the ν(C-H) out of plane vibration of the pyridine ring. The very strong bands at 1600 and 1400 cm^−1^ are due to asymmetric and symmetric vibrations of the acetate group, respectively [53]. The most characteristic ν(C-N), ν(C-C) ring vibration and bipyridine breathing modes can be observed in the ranges 1600.0–1608.9, 1533.4–1537.5, and 1004–1018.3 cm^−1^, respectively. These frequencies are shifted to higher values compared to free bipyridine indicating Zn coordination, which is confirmed by the ν(Zn-N) stretching vibration present as a faint peak at 515 cm^−1^ [53].

**Figure 7:**
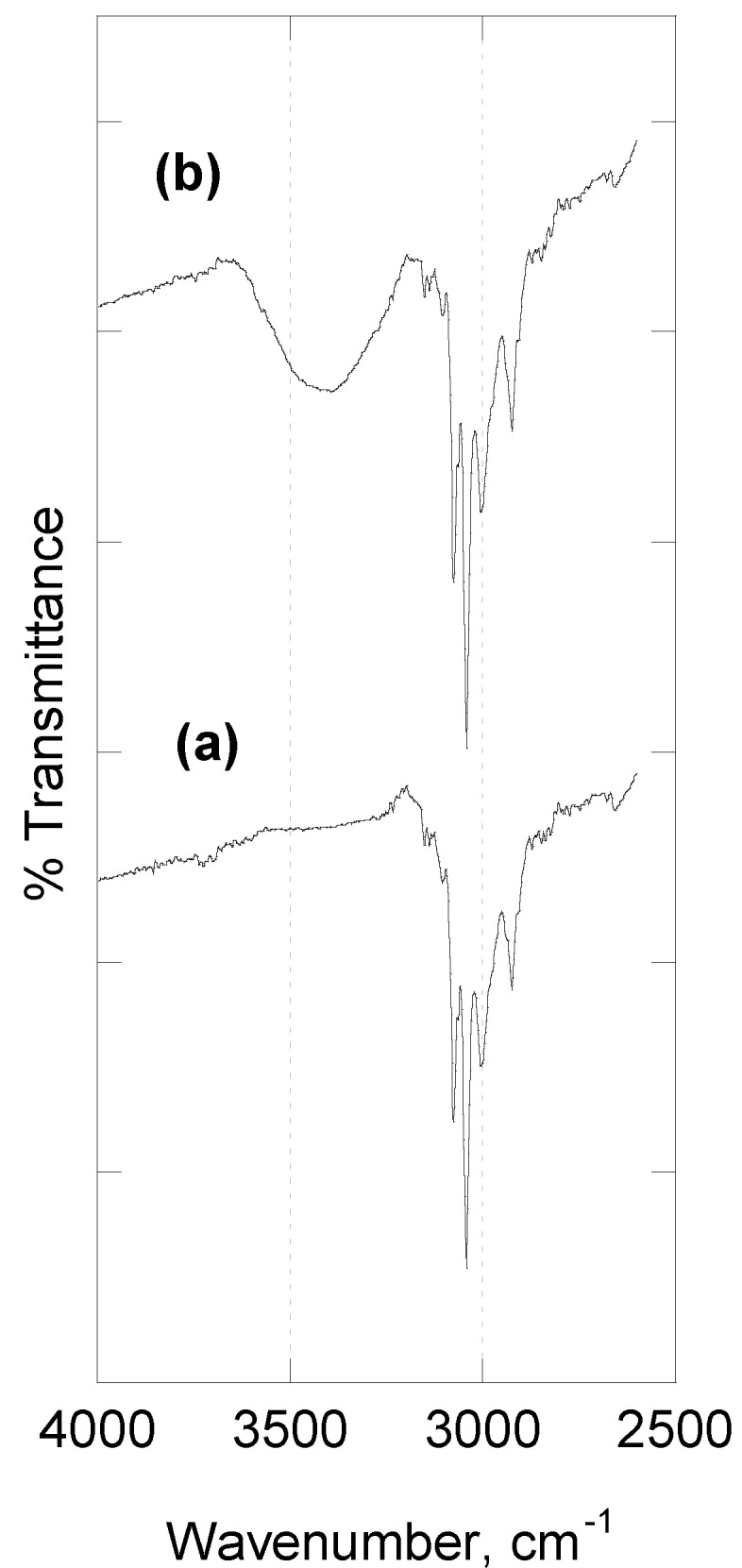
Magnification of FTIR spectra for (**a**) sample A and (**b**) sample B.

The above FTIR results are confirmed also for C and D samples. A magnification of sample B spectrum in the range 4000–2500 (Figure 7b) exhibits a large band at 3400 cm^−1^ indicating the presence of water. This band is not observed in sample A in Figure 7a, indicating that the second phase present in the sample B contains water in the structure.

**Figure 8:**
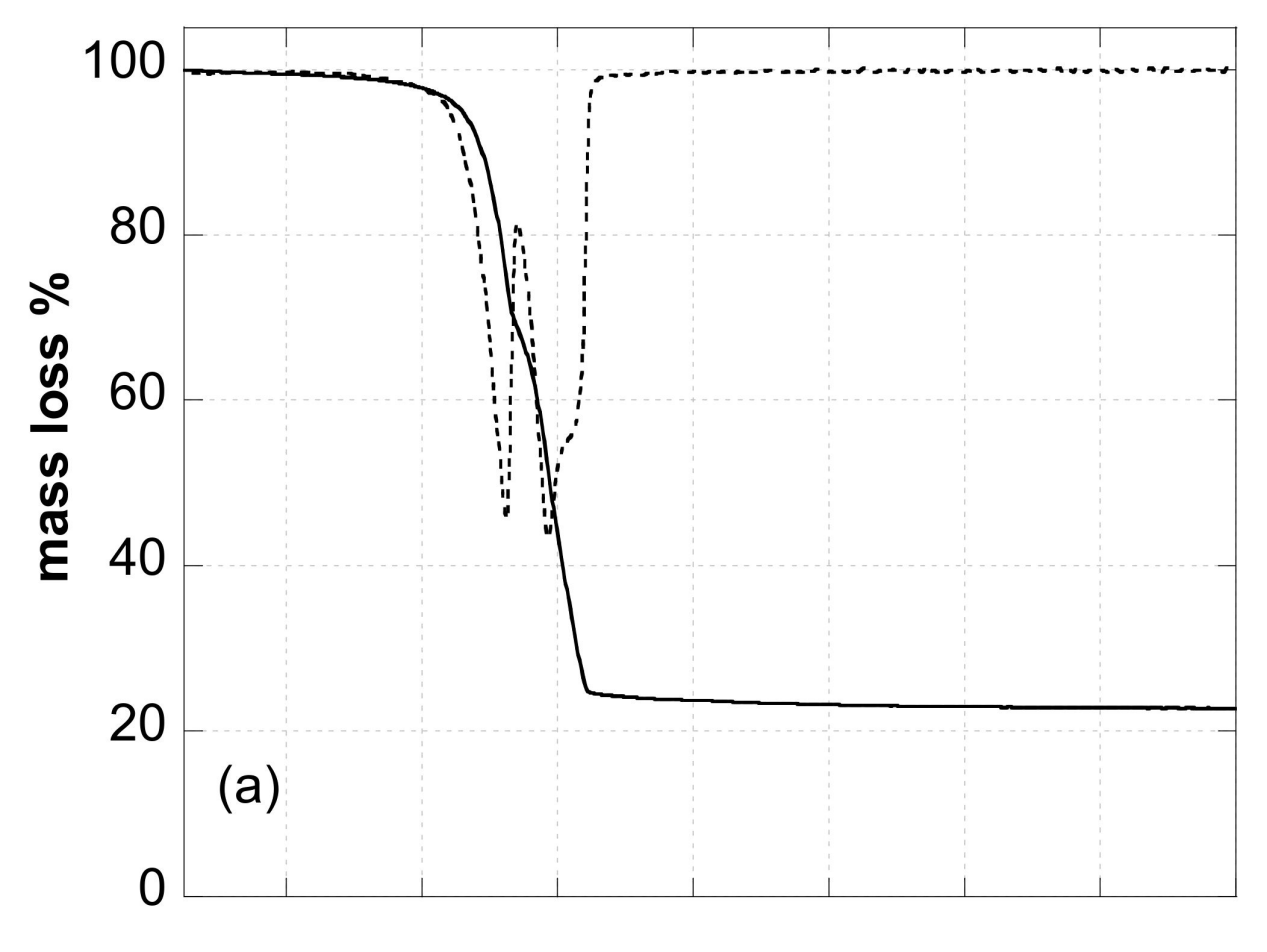

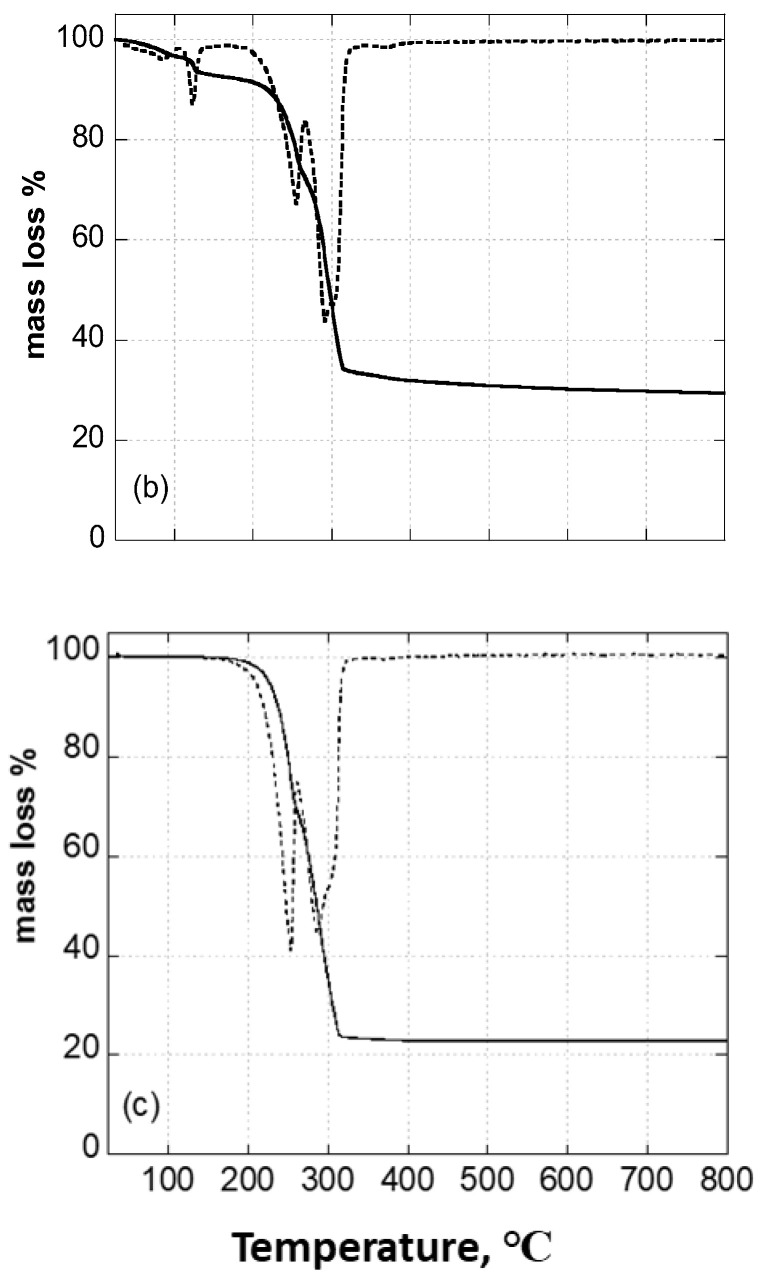
Thermogravimetric curves of *[Zn(4,4′-bipy)_2_(OAc)_2_]_∞_* samples prepared by the sonochemical process: (**a**) sample A; (**b**) sample B; (**c**) sample C.

In the thermogravimetric (TG) and the corresponding derivative (dTG) curves of Figure 8a–c, a main mass loss (and dTG peak) in the temperature range 165–325 °C is observed. Three slope changes are visible and the corresponding dTG peaks are centered at around 255 and 292 with a shoulder at 305 °C. This thermal behaviour can be ascribed to ligands decomposition, which gives rise to zinc oxide in all samples after the treatment at 800 °C. The related XRPD pattern is reported in the supporting information (Figure S5).

In sample A and C, no step in the 25–165 °C temperature range was observed. The remaining mass after 800 °C is around 23 wt%, in agreement with a 23.9 wt% theoretical value of complete decomposition of *[Zn(4,4′-bipy)_2_(OAc)_2_]_∞_* to ZnO [54]. The thermal properties of these two samples are almost indistinguishable (Figure 8a,c).

In sample B, two steps were observed in the temperature range 25–165 °C with the corresponding dTG peaks centered at 80 and 125 °C. These thermal losses are due to water elimination in agreement with XRPD and FTIR results. The overall remaining mass after thermal treatment at 800 °C is around 30 wt%, higher than the expected value of 23 wt%. The data agree with the CHN, XRPD, and FTIR analyses and confirm the presence of an amorphous secondary phase with a low content of carbon, hydrogen and nitrogen.

**Figure 9:**
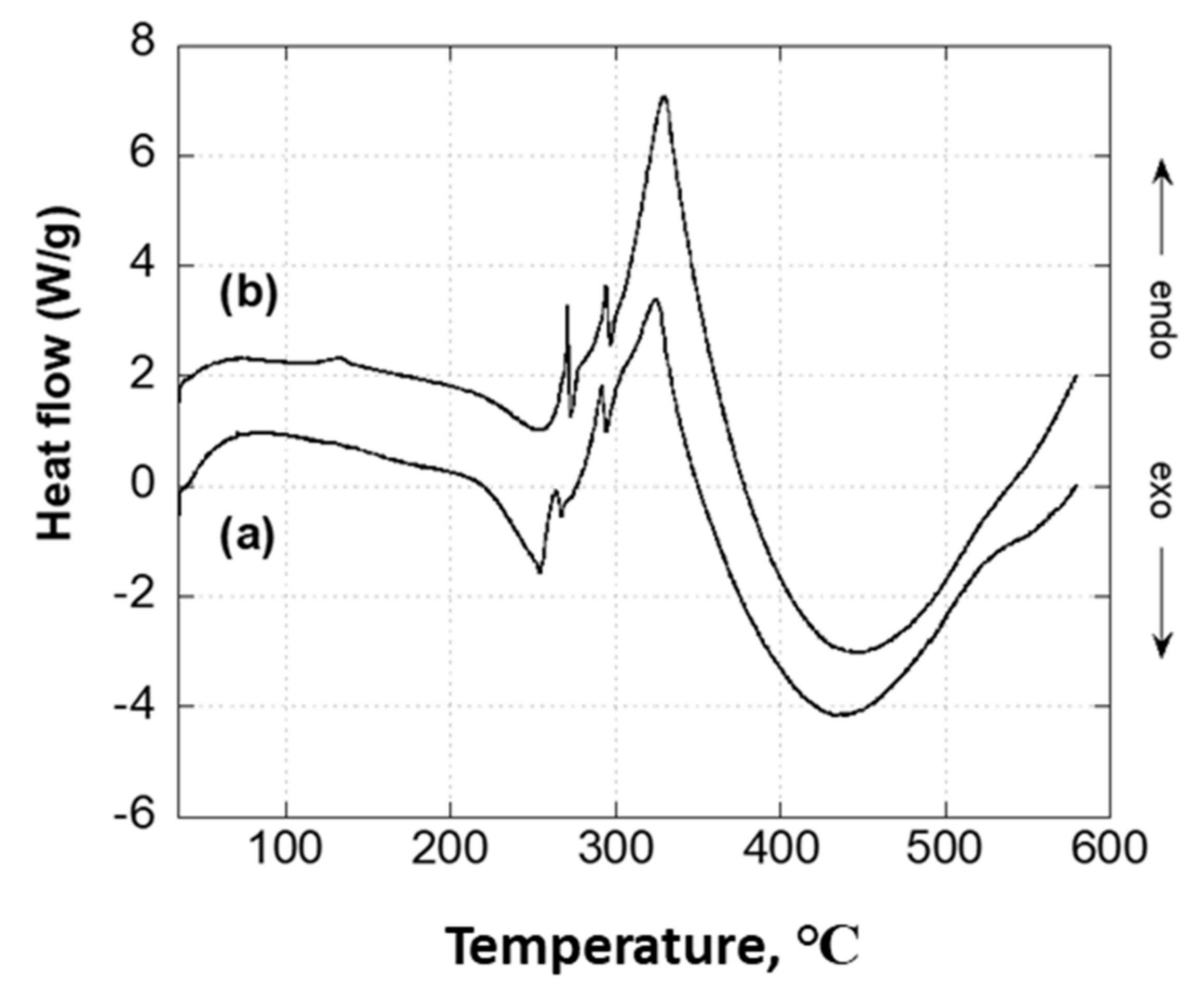
DSC curves of *[Zn(4,4′-bipy)_2_(OAc)_2_]_∞_* samples prepared by the sonochemical process: (**a**) sample A; (**b**) sample B.

The DSC curves for sample A and C, show three endothermic peaks centred around 255, 292 and 305 °C (Figure 9). For sample B an expected endothermic peak centred at 125 °C, due to water loss is also observed confirming the TG results.

Considering the above reported results, it is clear that the reactions of Zn^2+^ salts (chloride and acetate) with the 4,4′-bipyridine under sonochemical conditions allow the formation of the 1D coordination polymer. The reaction time is reduced to less than 5 min compared with several hours, avoiding the use of high temperatures of the reaction batch, with reaction yields comparable to those obtained by conventional synthesis. The acoustic cavitation process with high intensity ultrasonic energy induces extreme local conditions of high temperatures and pressure favouring the synthesis of coordination polymers.

### 2.3. Antimicrobial and Antibiofilm Activity

An initial evaluation using the Kirby–Bauer procedure showed that *Candida* spp., *Klebsiella pneumoniae*, and *Staphylococcus aureus* do not demonstrate sensitivity to the compounds (inhibition diameter ~ 0). On the contrary, the antimicrobial and antibiofilm analysis, evaluated in a liquid medium, reflected an appreciable activity; see Table 3. The inconsistency among the Kirby–Bauer and the antimicrobial and antibiofilm analysis results could be due to a steric hindrance in the agar structure so that it results in a diffusion block for the tested substances, as already experienced in our previous studies. In fact, both *[Zn(4,4′-bipy)Cl_2_]_∞_* and *[Zn(4,4′-bipy)_2_(OAc)_2_]_∞_* showed antimicrobial activity against the Gram-positive and Gram-negative model bacteria, independently from their difference in the cell structure, physiology, and metabolism, as well as against the pathogenic yeasts, except for *Candida krusei*. In this last context, *Candida albicans* resulted in the most sensitive yeast, in particular in the presence of *[Zn(4,4′-bipy)Cl_2_]_∞_*. Both coordination polymers showed MBC > 50 (> 4 μg/mL). In comparison with standard drugs evaluated in our laboratory and the data published in Eucast breakpoint data [55], these compounds showed antimicrobial activity against yeasts, the nosocomial pathogen *K. pneumoniae* and *S. aureus* MSRA. In fact, these strains had a multi-drug resistance profile, especially for azoles, fluconazole in primis for *Candida* spp., and some beta-lactams, cephalosporins, aminoglycosides, and macrolides for bacteria. In addition, these new compounds show an interesting antibiofilm activity (Table 3). In the bacterial strains, growth and biofilm inhibition were also both observed at 2 μg/mL. More commercial drugs are inactive or less active against microbial biofilm. This is due to a waterproof matrix. In particular, compound *[Zn(4,4′-bipy)Cl_2_]_∞_]* showed an MBIC of 2 ug/mL with *K. pneumoniae* and *S. aureus*, while they were less performant against Candida biofilm (MBIC > 4 μg/mL). Thus, this class of compounds led to promising future research and evaluations on new antimicrobials.

## 3. Materials and Methods

### 3.1. Chemicals and Reagents

Anhydrous zinc chloride, (ZnCl_2_, 98%, Aldrich, St. Louis, Missouri, US), anhydrous zinc acetate (Zn(CH_3_CO_2_)_2_, abbr. Zn(OAc)_2_, 98%, Aldrich), 4,4′-bipyridyne (abbr. 4,4′ Bipy, 98%, Aldrich), methanol (CH_3_OH, abbr. MeOH, C.Erba 99.9%), ethanol 99.8% (C_2_H_5_OH, A.C.S grade, J.T. Baker, Munich, DE), and ethanol 96% (C_2_H_5_OH, abbr. EtOH-96, A.C.S grade, J.T. Baker), dichloromethane (CH_2_Cl_2_, Aldrich 98%) were used without further purification. Fresh bidistillate milliQ water was used. Anhydrous ethanol (indicate as EtOH-100) was freshly prepared by distilling Ethanol 99.8% over calcium oxide (CaO, Aldrich 99.9%). For the microbiological assays, the microbial species (*Klebsiella pneumoniae,* DSM 681; *Staphylococcus aureus,* DSM 1104; *Candida albicans,* DSM 1386; *Candida krusei,* DSM 70075 *and Candida glabrata,* DSM 6425), microbial growth agar media (Muller Hinton agar and Sabouraud agar), Phosphate-buffered solution (PBS), crystal violet solution and DNAse free water solution were purchased from Microbiol (Uta, Cagliari, Italy). Sterile iron rivets, Ø 10 mm diameter and 2 mm thick, were from Firm (Milan, Italy).

### 3.2. Synthesis of [Zn(4,4′-bipy)Cl_2_]_∞_

**Scheme 1:**
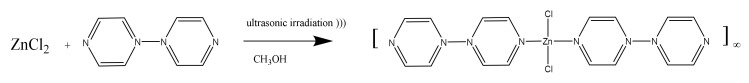
Reaction scheme of *[Zn(4,4′-bipy)Cl_2_]_∞_* sample.

The reaction between zinc (II) chloride and 4,4′-bipy to form *[Zn(4,4′-bipy)Cl_2_]_∞_* is shown in Scheme 1.

Two methanolic solutions (25 mL) of ZnCl_2_ (25 mM, 0.0852 g) and 4,4′-bipy (25 mM, 0.0976 g) were mixed. The obtained solution was sonicated using the Soniprep 150 ultrasonicator operating at 23 kHz adopting 10 cycles of 10 s sonication and 60 s rest. The obtained precipitate was centrifuged at 5500 rpm, washed with methanol, and allowed to dry at room temperature. White powder. Yield 63%. C_10_H_8_N_2_Cl_2_Zn (292.48): calcd. C 41.06, H 2.76, N 9.58; found C 40.74, H 1.87, N 10.71.

More experiments were carried out using water and methanolic solutions or water as the only solvent, obtaining very low yields (data not reported).

### 3.3. Synthesis of [Zn(4,4′-bipy)_2_(OAc)_2_]_∞_

**Scheme 2:**
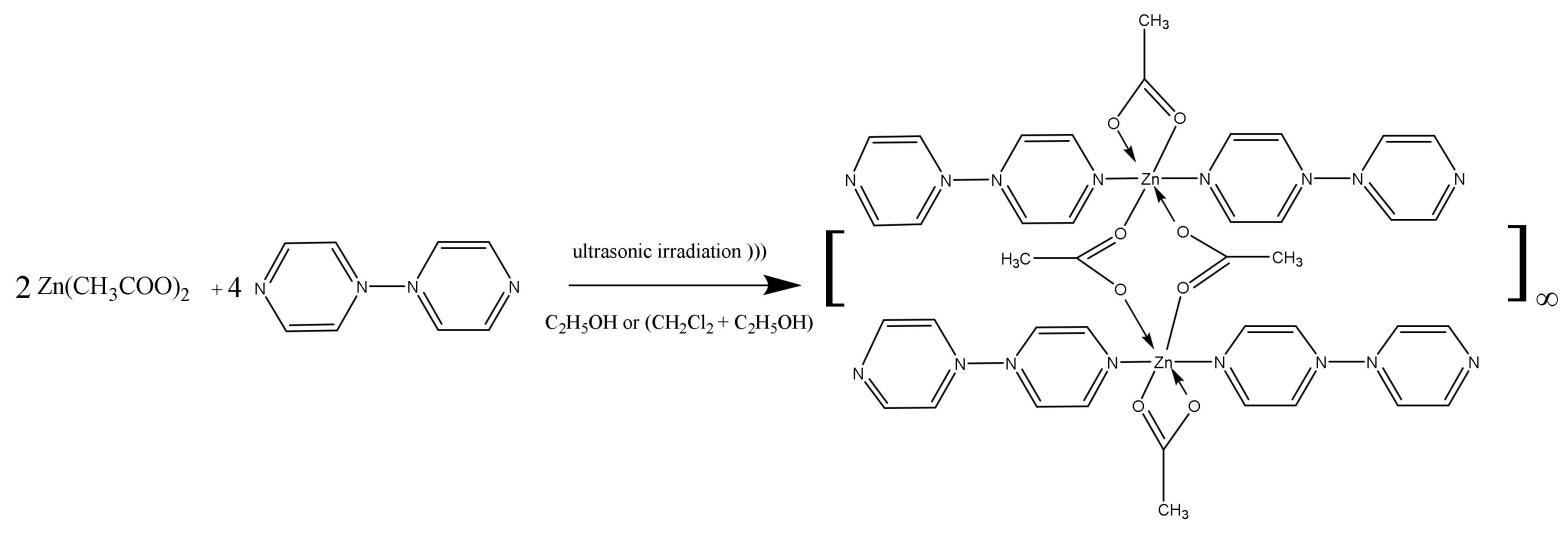
Reaction scheme of [*Zn(4,4′-bipy)_2_(OAc)_2_*]_∞_ sample.

The reaction between zinc (II) acetate and 4,4′-bipyridine to form *[Zn(4,4′-bipy)**_2_**(OAc)_2_]_∞_* is shown in Scheme 2.

Four different protocols (A, B, C, D) were used:

Sample (A), 15 mL of the EtOH-100 solution of Zn(OAc)_2_ (0.152 g, 0.83 mmol), was added to 2 mL of a CH_2_Cl_2_ solution of 4,4′-bipy (0.130 g, 0.83 mmol) and sonicated using the SONIPREP 150 ultrasonicator operating at 23 kHz and adopting 10 cycles of 10 s sonication and 60 s rest. The precipitate was filtered off, washed with EtOH-100, and allowed to dry at room temperature. The acronym of this sample is (sample A). White powder. Yield 78%. C_14_H_14_N_2_O_4_Zn (339.66): calcd. C 49.50, H 4.15, N 8.25; found C 49.11, H 3.22, N 9.41.

Sample (B), 15 mL of the ethanolic (96%) solution of Zn(OAc)_2_ (0.152 g, 0.83 mmol), was added to 2 mL of a CH_2_Cl_2_ solution of 4,4′-bipy (0.130 g, 0.83 mmol) and sonicated for 10 cycles of 10 s sonication and 60 s rest. The obtained precipitate was filtered off, washed with ethanol (96%), and allowed to dry at room temperature. This sample is indicated as (sample B). White powder. Yield 39%. C_14_H_14_N_2_O_4_Zn (339.66): calcd. C 49.50, H 4.15, N 8.25; found C 36.96, H 3.04, N 6.74.

Sample (C), the sample obtained by b) approach, was washed with methanol and allowed to dry at room temperature. The acronym of this sample is (sample C).

For sample (D), in order to perform a more eco-sustainable synthesis process, CH_2_Cl_2_ solvent was substituted in the ultrasonication process by ethanol as green solvent, and 15 mL of the aqueous solution of Zn(OAc)_2_ (0.152 g, 0.83 mmol) were added to 2 mL of a ethanolic (96%) solution of 4,4′-bipy (0.130 g, 0.83 mmol). Any precipitate was not observed after 24 h. Therefore, the solution was allowed to dry in the oven at 40°C for 24 h. The solid residue was washed with methanol and dried at room temperature. This sample has the acronym (sample D). White powder. Yield 64%. C_14_H_14_N_2_O_4_Zn (339.66): calcd. C 49.50, H 4.15, N 8.25; found C 49.36, H 3.20, N 9.70.

### 3.4. Physico-Chemical Characterization

#### 3.4.1. X-ray Powder Diffraction (XRPD)

XRPD analyses were performed by a Bruker D8 Advance diffraction system (daVinci design with TWIN optics) in the Bragg–Brentano focalizing geometry equipped with a CuKa source ((λ = 1.54056 Å), set at 40 mA and 40 kV, in the range of 5–80 (2ϑ degrees)) with a step of 0.030 2ϑ using LYNXEYE detector, and with an opportune counting time to optimize the signal/noise ratio. Rietveld structural refinement was performed using Maud software [56] to evaluate several parameters: phases contents, lattice parameter, average crystallite size, and microstrain. The instrument profile broadening was derived from the fitting of XRPD data obtained from standard samples [57].

#### 3.4.2. Fourier Transform Infrared Spectroscopy (FTIR)

FTIR analysis of the samples was carried out using a Bruker Tensor 27 spectrophotometer (Bruker, Billerica, Massachusetts, US), equipped with a diamond-ATR accessory and a DTGS detector; 128 scans at a resolution of 2 cm^−1^ were averaged from wavenumber 4000 to 400 cm^−1^.

#### 3.4.3. Thermal Analysis

Thermogravimetric analysis (TGA) was carried out at atmospheric pressure using a Perkin Elmer instrument TGA7 (Perkin Elmer, Waltham, Massachusetts, US) The measurements were performed under Ar flow (60 mL/min). Samples of 10 mg were placed in platinum crucibles and scanned in the temperature range of 30–800 °C with a heating rate of 10 °C/min. TGA7 instrument was calibrated with Curie points of Alumel, Nickel, Perkalloy, and Iron standard samples, and the temperature was obtained with an accuracy of ±1 °C.

Differential scanning calorimetry (DSC) measurements were carried out at atmospheric pressure using a Perkin Elmer instrument model DSC7 (Perkin Elmer, Waltham, Massachusetts, US). The measurements were performed under Ar flow (60 mL/min). Samples of 5 mg were placed in aluminum crucibles and scanned in the temperature range of 30–550 °C with a heating rate of 10 °C/min. DSC7 instrument was calibrated by measuring the melting temperature of metallic indium and zinc (99.999 mass % purity) and the temperature was obtained with an accuracy of ±0.1 °C.

#### 3.4.4. Elemental Analysis

The elemental analysis was also performed with a CHNS/O 2400-II series Perkin-Elmer instrument (Perkin Elmer, Waltham, Massachusetts, US).

### 3.5. Antimicrobial Assays

*[Zn(4,4′-bipy)Cl_2_]_∞_* and *[Zn(4,4′-bipy)_2_(OAc)_2_]∞* (sample D) were tested against a set of model bacteria *Klebsiella pneumoniae*, DSM 681 (Gram-negative) and Staphylococcus aureus, DSM1104 (Gram-positive) and yeasts *(Candida albicans*, DSM 1386; *Candida krusei*, DSM 70075; and *Candida glabrata*, DSM 6425).

As a first step, an agar diffusion test (Kirby–Bauer) was used to evaluate bacterial resistance/susceptibility to the *[Zn(4,4′-bipy)Cl_2_]_∞_* and *[Zn(4,4′-bipy)_2_(OAc)_2_]_∞_* suspended in a DNAse free water solution [58]. For each bacterium strain, 15 mL of agarized medium at 55 °C was added to a 90 mm Petri dish and, prior to agar solidification, four sterile iron rivets, Ø 10 mm diameter and 2 mm thick, were put into the agar mixture and then removed from the medium when it was cold. In these conditions a series of 5 wells were realized inside the agar thickness, which could contain 50 μL of the testing suspension [59]. Each strain was inoculated onto the plate surface using a sterile swab with bacterial 5 × 10^7^ (CFU) standardized inoculum. Three wells were used for the substance testing and two for a negative control. The Petri dishes were incubated in air at 37 °C for 24 h. After incubation, the diameter of the possible inhibition alone was measured, and the experiment was performed in triplicate. In this experiment, Muller–Hinton agar was used for the aerobic bacteria *Klebsiella pneumoniae* and *Staphylococcus aureus*, while *Sabouraud* agar was used for the yeasts *Candida albicans*, *Candida krusei,* and *Candida glabrata*.

The antimicrobial activity was performed in a liquid medium to calculate minimum inhibitory concentration and minimum bactericidal concentration, MIC and MBC, respectively. The experimental procedure was in accordance with EUCAST protocols, using the micro-broth dilution method with ½ serial dilutions in liquid medium [60]. For all compounds the tested concentration range was from 4 mg mL^−1^ to 0.039 mg mL^−1^. Each strain was inoculated in the exponential (log) growth phase by using a final concentration corresponding to 1 × 10^6^ CFU mL^−1^. After 24 h of growth at 37 °C, the turbidity of each set of combinations was measured by using a microplate reader Multiskan FC Microplate Photometer (Thermo Fisher Scientific IT, Milan, Italy) at λ = 620 nm. MIC was the lowest concentration that demonstrated the same negative control absorbance, while MBC represented the lowest concentration able to reduce 99.9% of bacteria vitality (CFU mL^−1^), when the microbial suspension was plated in agar medium.

Minimal biofilm inhibitory concentration (MBIC) for all compounds was evaluated following the crystal violet staining protocol described by Montana University Center for Biofilm Engineering [61] and described by different authors [62]. Briefly, in a multi-well plate containing 1 × 10^6^ CFU mL^−1^ of bacterial/yeast suspension, a serial dilution from 50% to 0.05% were evaluated (4–0.039 mg mL^−1^), these cultures were then maintained at 37 °C for 3 days. After incubation, the plates were washed three times with PBS, and the biofilm adhering to the well surface was stained with a 0.4% crystal violet solution for 2 min. Following two washes with PBS, 100 μL of 30% acetic acid to each well was then added. The biofilm was measured by a colorimetric assay at 620 nm by Multiskan FC Microplate Photometer (Thermo Fisher Scientific IT, Milan). For each formulate, the MBIC was the lowest concentration reporting an absorbance comparable with negative control (medium without bacteria).

Standard drugs (a set of commercial antibiotics and antimycotics) were used for a comparative evaluation with our compounds [63,64]. The primary data are available in the supplementary materials session [65,66]. The antibiogram was performed in accordance with the EUCAST protocol and obtained by using the Vitek-2 Compact system (bioMérieux France) in accordance with manufacturer instructions, Table S1 and Figure S6, Supporting information).

## 4. Conclusions

Two stable polymorphs of *[Zn(4,4′-bipy)Cl_2_]_∞_* CPs were obtained in the form of nanocrystals by ultrasonication starting from zinc chloride and 4,4′-bipyridine in a green reaction media. The Zn^2+^ ion forms a distorted tetrahedral geometry, and the coordination type is a one-dimensional zigzag infinite chain.

In the synthesis with zinc acetate, nanocrystals of *[Zn(4,4′-bipy)_2_(OAc)_2_]_∞_* CPs were obtained according to four different procedures by ultrasonication. In this case, the Zn^2+^ ion exhibits a pseudo-octahedral geometry and the overall coordination type is a distinctive one-dimensional double-chain structure. When in the reaction batch water amount was significantly present, a secondary unknown phase was also obtained. However, such a phase can be removed by washing the as-prepared sample with methanol.

To the best of our knowledge, our study is the first that demonstrates the antimicrobial and antibiofilm activity of *[Zn(4,4′-bipy)Cl_2_]_∞_* and *[Zn(4,4′-bipy)_2_(OAc)_2_]_∞_* CPs against *Candida* spp. yeasts, Gram-negative and Gram-positive bacteria.

In conclusion, the sonochemical synthetic protocol presented in this paper can be considered a valid technique to replace conventional synthesis for the preparation in a fast, simple, and green way of CPs as effective materials for several applications. Our results can represent a first step toward the production according to green chemistry principles of CPs with a broad range antimicrobial activity. The next step will be to understand the mode of action involved in the antimicrobial and antibiofilm activity on the different microbial species.

## Figures and Tables

**Table 1:** Summary of X-ray powder data and structure refinement parameters for compounds obtained by sonochemical method.

Compounds	[C_10_ H_8_ Cl_2_ N_2_ Zn]_∞_—Polymorph I	[C_10_ H_8_ Cl_2_ N_2_ Zn]_∞_—Polymorph II
**Crystal system**	monoclinic	orthorhombic
**Space group**	C2/c (n. 15)	Pnma (n. 62)
**a/Å**	15.80(1)	17.37(1)
**b/Å**	5.11(1)	12.49(1)
**c/Å**	14.58(1)	5.10(1)
**a/°**	90	90
**b/°**	110.24(1)	90
**g/°**	90	90
**Z**	4	4
**wt%**	48.0(1)	52.0(1)
$R_{wp}=100\cdot \sum_{i=1}^{n}w_{i}\;{\left(I_{i}^{exp}-I_{i}^{calc}\right)}^{2}/\sum_{i=1}^{n}{\left(w_{i}I_{i}^{exp}\right)}^{2}=15.0\%$		

**Table 2:** Summary of X-ray powder data and structure refinement parameters for compound *[Zn(4,4′-bipy)2(OAc)2]_∞_* obtained by sonochemical method (<D> average crystal size; σ microstrain). For comparison, single-crystal XRD data of samples obtained by conventional synthesis are reported [51].

Compound	*[Zn(4,4′-bipy)_2_(OAc)_2_]* *_∞_*	*[Zn(4,4′-bipy)_2_(OAc)_2_]* *_∞_*
**XRD method**	powder	single crystal
**Crystal system**	triclinic	triclinic
**Space group**	P-1	P-1
**a/Å**	8.10(1)	8.088(1)
**b/Å**	9.18(1)	9.190(1)
**c/Å**	10.64(1)	10.676(1)
**α/°**	109.56(5)	109.70(1)
**β/°**	100.13(5)	100.03(1)
**γ/°**	101.82(5)	101.78(1)
**Z**	2	2
**T/K**	298	283–303
**ϑ min-max/°**	5–80	9.5–10.5
**<D>/nm**	102(5)	-
**σ/%**	< 0.1	-
**Wavelength/Å**	1.54056 (CuKα)	0.71069 (MoKα)
**R indices (all data)**	R_wp_% = 10.0	R_1_ = 0.0278; wR_2_ = 0.0732
$w^{-1}=\left[\sigma^{2}\left(F_{exp}^{2}\right)+{\left(0.0320P\right)}^{2}+2.1046P\right]\;where$		
$P=\left[F_{exp}^{2}+2F_{calc}^{2}\right]$;		
$R_{1}=\sum_{i=1}^{n}\left(\left|F_{exp,i}\right|-\left|F_{calc,i}\right|\right)/\sum_{n=1}^{\infty }\left|F_{exp,i}\right|$;		
$wR_{2}={\left\{\sum_{i=1}^{n}\left[w{\left(F_{exp,i}^{2}-F_{calc,i}^{2}\right)}^{2}\right]/\sum_{n=1}^{\infty }[w{\left(F_{exp,i}^{2}\right)}^{2}]\right\}}^{\frac{1}{2}}$;		
$R_{wp}=100\cdot \sum_{i=1}^{n}w_{i}\;{\left(I_{i}^{exp}-I_{i}^{calc}\right)}^{2}/\sum_{i=1}^{n}{\left(w_{i}I_{i}^{exp}\right)}^{2}$.		

**Table 3:** Percentage dilution values for MIC, MBC, MBIC calculated for each tested compound.

Strain	*[Zn(4,4′-bipy)Cl_2_]_∞_*			*[Zn(4,4′-bipy)_2_(OAc)_2_]_∞_*		
	* MIC	* MBC	* MBIC	* MIC	* MBC	* MBIC
*Candida albicans* (DSM 1386)	25	>50	25	6.25	>50	6.25
*Candida krusei* (DSM 70075)	>50	>50	>50	>50	>50	>50
*Candida glabrata* (DSM 6425)	>50	>50	>50	25	>50	25
*Klebsiella pneumoniae* (DSM 681)	25	>50	25	25	>50	25
*Staphylococcus aureus* (DSM 1104)	25	>50	25	25	>50	25

* (% dilution).

## Data Availability

Not applicable.

## Supplementary Materials

The following supporting information can be downloaded at: https://www.mdpi.com/article/10.3390/molecules27196677/s1, Figure S1: *[Zn(4,4**′-bipy)Cl2]**∞* UBOCIX02 molecular structure: green stick (Chlorine), blue (Nitrogen). a) View down the crystallographic a axis and b) View down the crystallographic b axis.; Figure S2: *[Zn(4,4**′-bipy)Cl2]**_∞_* UBOCIX03 molecular structure; green stick (Chlorine), blue (Nitrogen). a) View down the crystallographic a axis and b) View down the crystallographic b axis; Figure S3: Rietveld refinement for sample A: experimental data (dots), simulated curve (solid lines) and curve difference (dashed lines). Lower vertical bars represent reflection positions of *[Zn(4,4**′bipy)2(OAc)2]**_∞_* phase obtained by single crystal data [51]; Figure S4: *[Zn(4,4′-bipy)2(OAc)2]**_∞_* molecular structure. Red stick (Oxygen), Blue (Nitrogen). View down the crystallographic b axis; Figure S5: XRPD data of sample A thermally treated at 800 °C. Lower vertical marks represent reflection positions of ZnO phase [54]; Figure S6: MIC values measured with three different multidrug resistant (MDR) Candida species used in this study. Methods in according with EUCAST guidelines [65,66]; Table S1: Drug susceptibility profile whitin the bacterial strains used in this work as control.
