# Supplementary material for: Green Preparation of Antimicrobial 1D-Coordination Polymers: [Zn(4,4′-bipy)Cl2]∞ and [Zn(4,4′-bipy)2(OAc)2]∞ by Ultrasonication of Zn(II) Salts and 4,4′-Bipyridine
Source: Molecules. 2022 Oct 7;27(19):6677. doi: 10.3390/molecules27196677 (PMC9572912; doi:10.3390/molecules27196677)
Supplement: Supplementary file 1 [file molecules-27-06677-s001.zip › molecules-1871484-supplementary.pdf]

Supplemental Materials

Green Preparation of Antimicrobial 1D-Coordination Polymers: *[Zn(4,4′-bipy)Cl₂]∞* and *[Zn(4,4′-bipy)₂(OAc)₂]∞* by Ultrasonication of Zn(II) Salts and 4,4′-Bipyridine

a

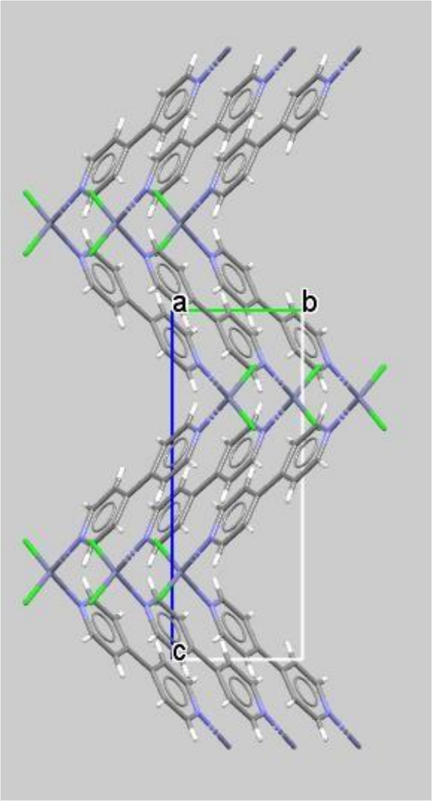

b

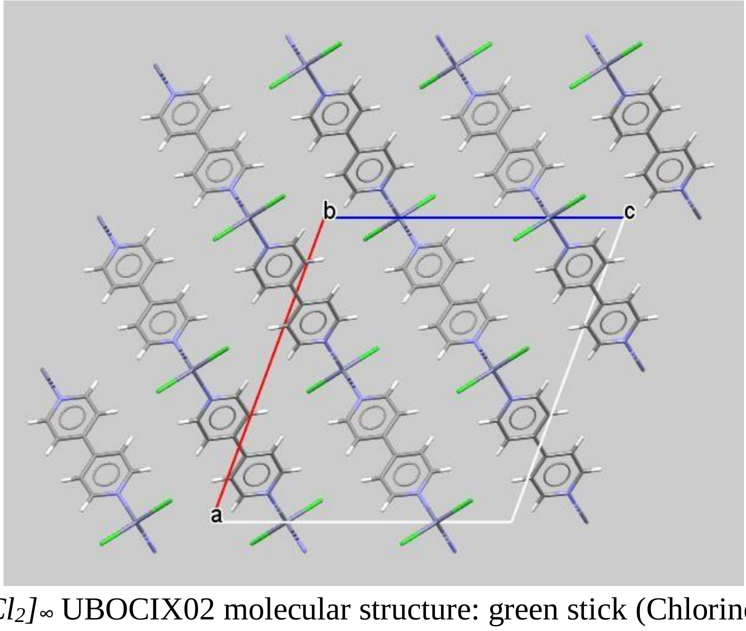

Figure S1. *[Zn(4,4’-bipy)Cl$_2$]$_\infty$* UBOCIX02 molecular structure: green stick (Chlorine), blue (Nitrogen). (**a**) View down the crystallographic a axis and (**b**) View down the crystallographic b axis.

a

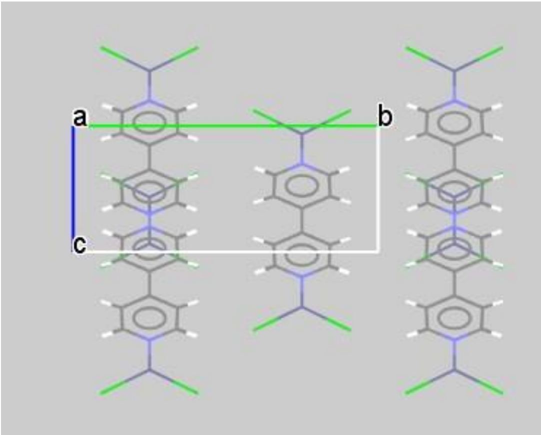

b

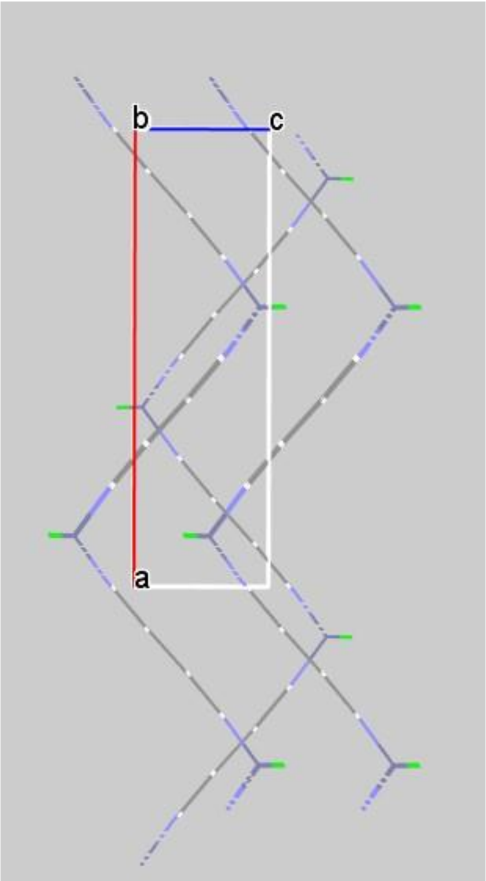

Figure S2. *[Zn(4,4'-bipy)Cl$_2$]$_\infty$* UBOCIX03 molecular structure; green stick (Chlorine), blue (Nitrogen). (**a**) View down the crystallographic a axis and (**b**) View down the crystallographic b axis.

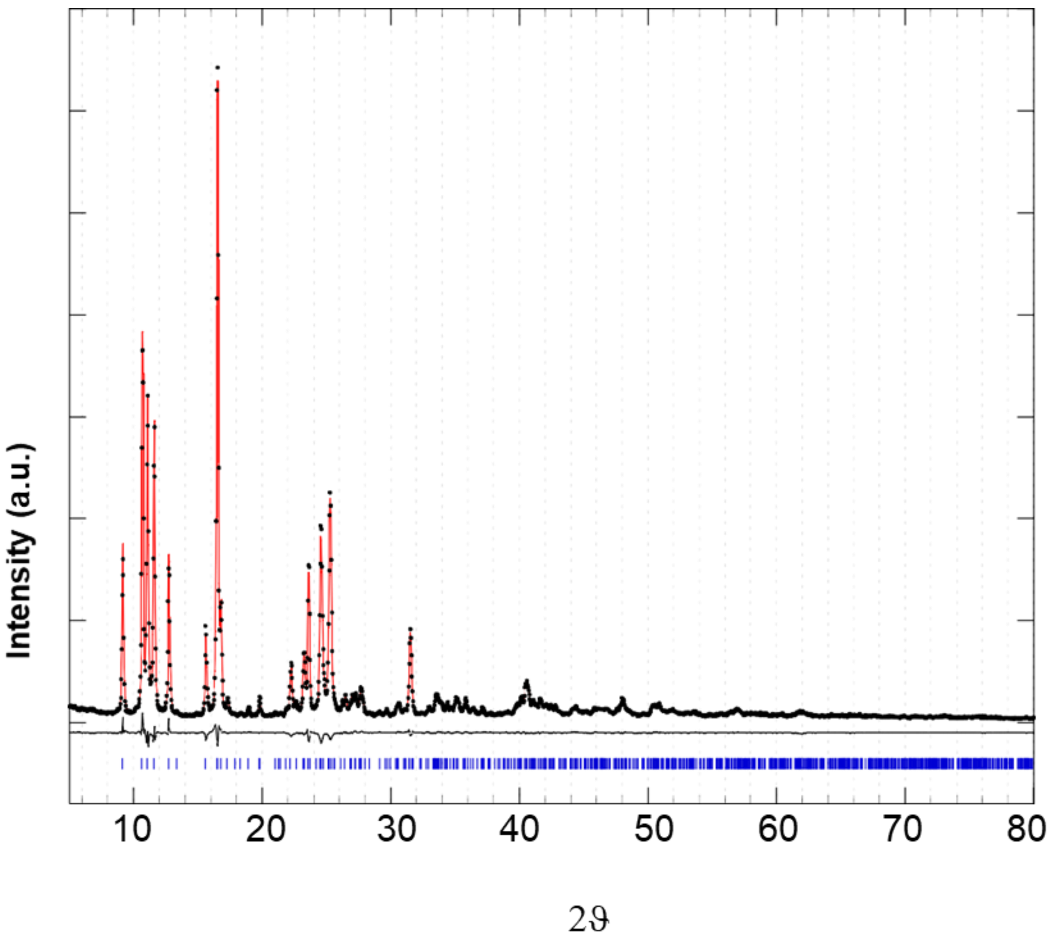

Figure S3. Rietveld refinement for sample A: experimental data (dots), simulated curve (solid lines) and curve difference (dashed lines). Lower vertical bars represent reflection positions of *[Zn(4,4'bipy)$_2$(OAc)$_2$]$_\infty$* phase obtained by single crystal data. [25].

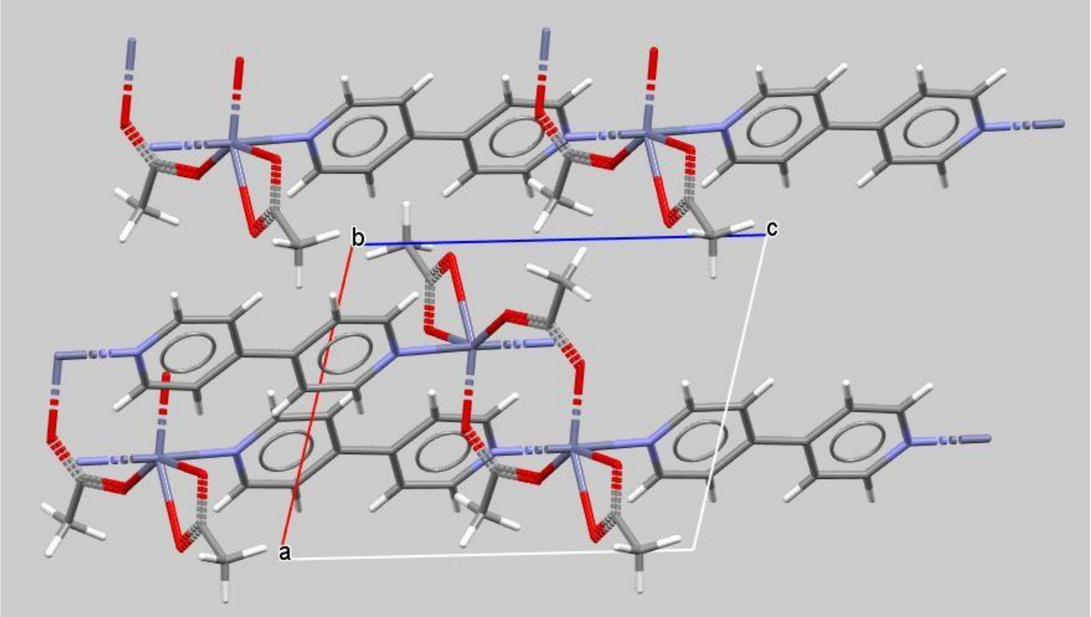

Figure S4. *[Zn(4,4'-bipy)$_2$(OAc)$_2$]$_\infty$* molecular structure. Red stick (Oxygen), Blue (Nitrogen). View down the crystallographic b axis.

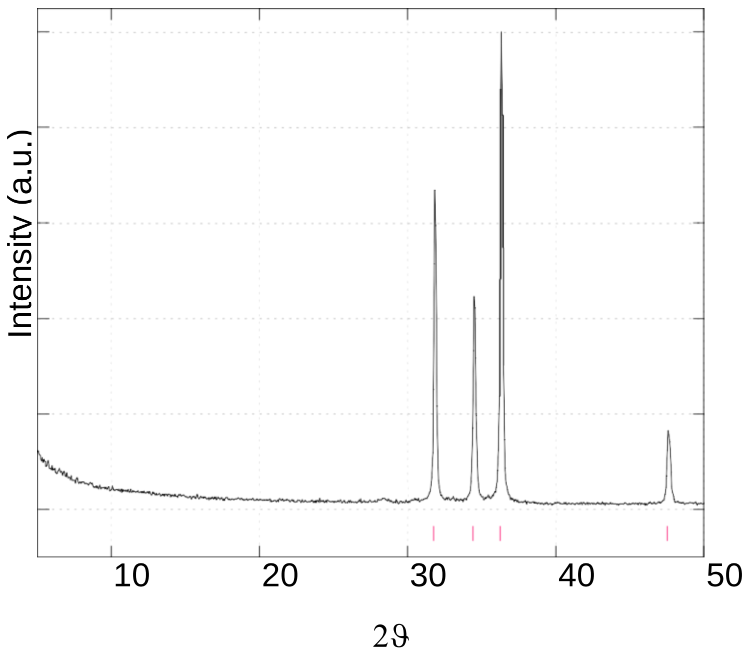

Figure S5. XRPD data of sample A thermally treated at 800°C. Lower vertical marks represent reflection positions of ZnO phase [24].

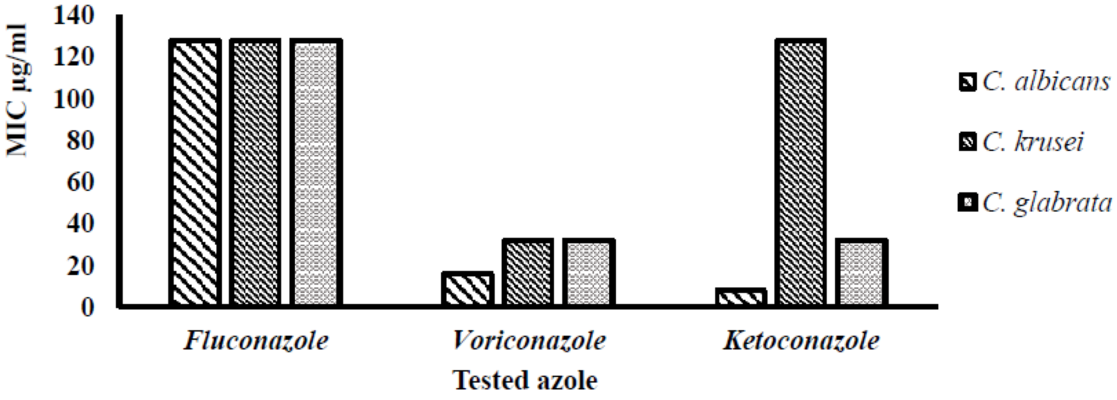

Figure S6. MIC values measured with three different multidrug resistant (MDR) Candida species used in this study. Methods in according with EUCAST guidelines [63,64]

Table S1. Drug susceptibility profile whitin the bacterial strains used in this work as control**.**

Tested Drug	*K. pneumoniae*	*S. aureus**
Amikacin	**R**	-
Amoxicillin/ac. Clavulanic	**R**	-
Benzylpenicillin	-	**R**
Cefepime	-	-
Cefotaxime	S	-
Ceftaroline	-	S
Ceftazidime	**R**	-
Ciprofloxacin	-	-
Clindamycin	-	S
Colistin	-	-
Daptomycin	-	S
Ertapenem	S	-
Erythromycin	-	**R**

Fosfomycin	-	-
Fusidic acid	-	S
Gentamicin	-	S
Imipenem	-	-
Levofloxacin	-	S
Linezolid	-	S
Meropenem	**R**	-
Oxacillin	-	S
Piperacillin-tazobactam	S	-
**Rifampicin**	-	S
Teicoplanin	-	S
Tetracycline	-	S
Tigecycline	-	S
Trimethoprim-sulfamethoxazole	-	S
Vancomycin	-	S

Legend: drug susceptibility profile in according with AES EUCAST_Jannuary_2017 MIC values, (R = resistant, S= Sensitive), Methods in according with EUCAST guidelines, EUCAST: http://www.eucast.org/ast_of_bacteria/guidance_documents/ [63–68]. * = Methicillin resistant (MSRA) Strain. Antibiotic susceptibility was determined by Vitek-2 Compact system.
